# 2D Polymer Nanosheets for Membrane Separation

**DOI:** 10.1002/advs.202103814

**Published:** 2022-01-27

**Authors:** Fei Wang, Zhao Zhang, Imran Shakir, Chengbing Yu, Yuxi Xu

**Affiliations:** ^1^ School of Materials Science and Engineering Shanghai University Shanghai 201800 China; ^2^ School of Engineering Westlake University Hangzhou Zhejiang Province 310024 China; ^3^ School of Engineering Westlake Institute for Advanced Study Hangzhou Zhejiang Province 310024 China; ^4^ Department of Materials Science and Engineering University of California Los Angeles CA 90095 USA; ^5^ Sustainable Energy Technologies Center College of Engineering King Saud University Riyadh 11421 Saudi Arabia

**Keywords:** 2D polymer nanosheets, membrane fabrication, membrane separation application, structure‐property relationship, synthetic method

## Abstract

Since the discovery of single‐layer graphene in 2004, the family of 2D inorganic nanosheets is considered as ideal membrane materials due to their ultrathin atomic thickness and fascinating physicochemical properties. However, the intrinsically nonporous feature of 2D inorganic nanosheets hinders their potential to achieve a higher flux to some extent. Recently, 2D polymer nanosheets, originated from the regular and periodic covalent connection of the building units in 2D plane, have emerged as promising candidates for preparing ultrafast and highly selective membranes owing to their inherently tunable and ordered pore structure, light weight, and high specific surface. In this review, the synthetic methodologies (including top–down and bottom–up methods) of 2D polymer nanosheets are first introduced, followed by the summary of 2D polymer nanosheets‐based membrane fabrication as well as membrane applications in the fields of gas separation, water purification, organic solvent separation, and ion exchange/transport in fuel cells and lithium‐sulfur batteries. Finally, based on their current achievements, the authors’ personal insights are put forward into the existing challenges and future research directions of 2D polymer nanosheets for membrane separation. The authors believe this comprehensive review on 2D polymer nanosheets‐based membrane separation will definitely inspire more studies in this field.

## Graphical abstract

In this review, the preparation of 2D polymer nanosheets and polymer nanosheets‐based membranes are first introduced, followed by the summary of diverse membrane applications in gas separation, water purification, organic solvent separation, and ion exchange/transport. Finally, the challenges and future research directions of 2D polymer nanosheets‐based membranes separation are presented.

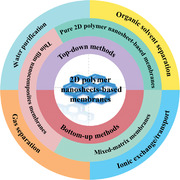

## 1 Introduction

Membrane‐based separation technology has attracted considerable attention since late 1960s. Taking advantage of high separation efficiency, low energy consumption, and easy scale‐up, membrane‐based separation technology has successfully applied in many separation fields, such as gas separation, desalination, water treatment, and so on.^[^ ^1^, ^2^, ^3^ ^]^ Advanced membranes with extraordinary perm‐selectivity are essential to the development of membrane‐based separation technology. Currently, most commercial membranes are fabricated from amorphous polymers such as polysulfone (PSf), poly(vinylidene fluoride) (PVDF), polyacrylonitrile (PAN), polydimethylsiloxane, and highly cross‐linked polyamide materials, which usually suffer from a well‐known “trade‐off” limit between permeability and selectivity.^[^ ^4^ ^]^ Although the addition of nanoparticles into the polymeric membrane matrix has improved this issue to some extent, the poor interfacial compatibility between inorganic nanofillers and polymers may lead to the formation of defects as non‐selective channels, posing a tough difficulty to achieve a high selectivity. On the other hand, the feature of the intrinsically disordered structure of amorphous polymers makes it difficult to achieve precise molecule sieving from different solutes with similar molecular size. Therefore, it is highly desirable to develop novel advanced membrane materials with fast and precise ionic/molecular transport.

Since the successful discovery of single‐layer graphene exfoliated from graphite by Geim in 2004,^[^ ^5^ ^]^ the family of 2D materials has been considered attractive candidates for membrane separations due to their fascinating physical and chemical properties. After that, a series of novel inorganic 2D materials including graphene oxide (GO),^[^ ^6^ ^]^ Mxenes,^[^ ^7^ ^]^ transition metal dichalcogenides,^[^ ^8^ ^]^ layered double hydroxides have been synthesized.^[^ ^9^ ^]^ Generally, such 2D materials‐based membranes are constructed by assembling 2D nanosheets into lamellar membranes using vacuum‐assisted filtration processes, with interlayer galleries used to provide molecular transport passages. Because of their ultrathin thickness, tunable transport channels by adjusting the interlayer spacing as well as the chemical and mechanical robustness, these 2D inorganic materials‐based membranes show outstanding size‐selective permeation properties. Unfortunately, despite of their fascinating membrane separation properties, the intrinsically nonporous feature and the resulting overlong permeation pathways along the nanosheet interlayers severely hinder their potential to achieve a higher flux. Although pores in these 2D materials have been made successfully through drilling with chemical etching or electron beams,^[^ ^10^, ^11^ ^]^ the pore‐forming conditions are too harsh and far from practical application. Therefore, novel and revolutionary 2D materials with intrinsically porous structures are extremely needed.

**Figure 1:**
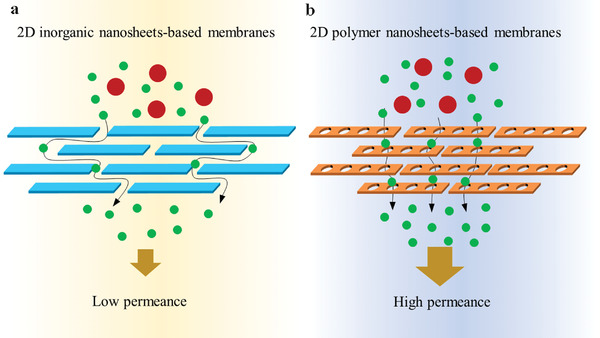
a) The model of the lamellar membrane composed of 2D inorganic nanosheets in which the mass transport could be achieved along the nanosheet interlayers (horizontal transport). b) The schematic diagram for mass transport across the 2D polymer nanosheets‐based membranes along the 1D porous nanochannels in the vertical direction.

2D polymer nanosheets, which are composed of repeat units with internal periodicity in 2D plane, have recently received great interest.^[^ ^12^ ^]^ In comparison with traditional inorganic 2D materials, 2D polymer nanosheets show the advantages of light weight, good flexibility, excellent structural controllability and designability, and facilely‐tailored functionality, thus attracting increasing interests in the field of gas storage, adsorption, catalysis, sensing, energy storage, and conversion.^[^ ^13^, ^14^ ^]^ More importantly, the regular and periodic connection of the building units in 2D plane endows 2D polymer nanosheets with uniform and orderly arranged pores, and the pore size can be chemically designed and tuned by selecting building units. These unique and fascinating characteristics make 2D polymer nanosheets promising candidates for constructing ultrafast and highly‐selective membranes. As shown in **Figure** **1**, the inherently tunable and ordered pore structure of 2D polymers could offer shorter permeation pathways and more precise molecular‐level sieving than traditional nonporous 2D materials. Over the past several years, a remarkable progress has been made on the design and fabrication of 2D polymer nanosheets‐based membranes for multiple applications including gas separation membranes, liquid separation membranes in water and organic solvent systems, and ion exchange/transport membranes in fuel cells and batteries. However, the related review of the progress is still very scarce but highly desired for inspiring more efforts and advancing the further development in this field.

**Figure 2:**
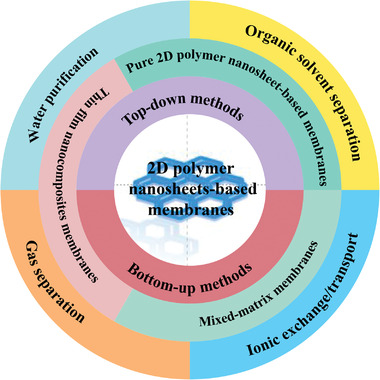
The scope of this review.

In this review, we first introduce the synthetic approaches of 2D polymer nanosheets including top–down and bottom–up methods. Next, we summarize the current state‐of‐the‐art 2D polymer nanosheets‐based membranes, including representative pure 2D polymer nanosheets‐based membranes, mixed‐matrix membranes (MMMs), and thin film nanocomposite membranes and their corresponding fabrication strategies. Subsequently, the applications of 2D polymer nanosheets‐based membranes in the fields of gas separation, organic solvent separation, water purification, and ionic exchange/transport in fuel cells and lithium‐sulfur batteries are discussed in detail. Finally, we put forward our own perspectives on the present challenges and the future development directions of 2D polymer nanosheets for membrane separation. The scope of this review is shown in **Figure** **2**.

## 2 Preparation of 2D Polymer Nanosheets

As for 2D polymer nanosheets, they generally refer to the covalent‐bonded polymer sheets with in‐plane periodicity and single‐layer or few‐layer thickness. According to this definition, 2D polymer nanosheets include 2D covalent organic framework (COF) nanosheets, 2D graphitic carbon nitride (g‐C_3_N_4_) nanosheets, 2D covalent triazine framework (CTF) nanosheets, and so on. These nanosheets not only possess similar planar, flexible, and freestanding characteristics to graphene, but also have the advantages of excellent structural designability and controllability. So far, a number of synthetic methods for producing ultrathin 2D polymer nanosheets have been discovered. The precise and efficient synthesis of high‐quality 2D polymer nanosheets is of great importance for further research on their physicochemical properties and various potential applications including membrane separation. In this section, we mainly discuss two kinds of strategies for preparing 2D polymer nanosheets including top–down and bottom–up strategies.

### 2.1 Top–Down Methods

Most of bulk 2D polymer materials are typical layered structure, in which the neighboring layers are usually stacked via relatively weak interactions (van der Waals forces, *π*–*π* stacking, hydrogen bonding, etc.).^[^ ^15^ ^]^ The top–down strategy usually utilizes external forces to directly break the weak interactions between the adjacent layers of polymers so that multi‐/mono‐layer polymer nanosheets can be successfully achieved. So far, the well‐developed exfoliation methods generally include the liquid phase exfoliation (LPE),^[^ ^16^, ^17^, ^18^, ^19^ ^]^ chemical exfoliation,^[^ ^20^, ^21^, ^22^, ^23^ ^]^ and mechanical exfoliation,^[^ ^24^, ^25^, ^26^ ^]^ all of which have been used to obtain ultrathin 2D polymer nanosheets.

**Figure 3:**
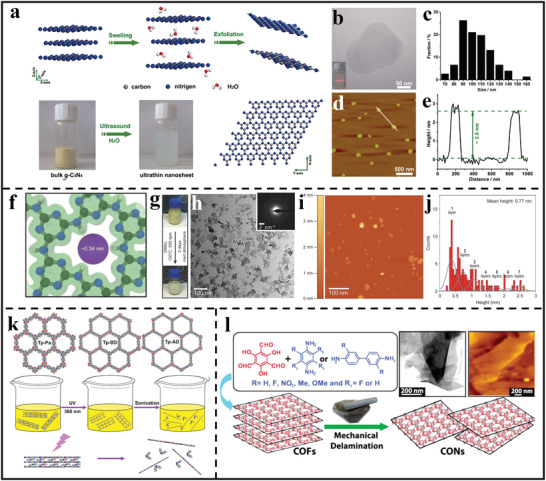
Representative types of the exfoliation approaches through various top–down methods. a) Schematic illustration of exfoliating g‐C_3_N_4_ into nanosheets through LPE method. b) TEM image of g‐C_3_N_4_ nanosheet, inset is the Tyndall effect of the g‐C_3_N_4_ nanosheet aqueous solution. c) Size distribution of synthesized g‐C_3_N_4_ nanosheets. d) AFM image and e) corresponding height image of the synthetic g‐C_3_N_4_ nanosheets. (a–e) Reproduced with permission.^[^ ^19^ ^]^ Copyright 2013, American Chemical Society. f) Structure of the PTI layer; C, N, and H atoms are colored in gray, blue, and white, respectively. g) Photographs of the PTI dispersion before and after exfoliation. h) Bright‐field TEM image of the dispersion of PTI nanosheets, inset shows a SAED pattern of the PTI nanosheet. i) AFM image and j) corresponding height histogram of PTI nanosheets. (f–j) Reproduced with permission.^[^ ^28^ ^]^ Copyright 2020, American Association for the Advancement of Science. k) Illustration of exfoliating bulk COFs into few‐layer 2D polymer nanosheets through azobenzene‐assisted chemical exfoliation method. Reproduced with permission.^[^ ^33^ ^]^ Copyright 2018, Elsevier. l) Formation of *β*‐Ketoenamine‐based COF nanosheets from bulk COFs through mechanical exfoliation and corresponding TEM and AFM images of COF nanosheets. Reproduced with permission.^[^ ^36^ ^]^ Copyright 2013, American Chemical Society.

By virtue of the LPE method, a typical example of 2D polymer nanosheets was achieved through exfoliating g‐C_3_N_4_ bulk materials.^[^ ^19^ ^]^ As shown in **Figure** **3**a, through simple sonication in water to break the weak interlayer interactions of g‐C_3_N_4_, nearly transparent ultrathin g‐C_3_N_4_ nanosheets have been successfully obtained, showing a size distribution in the range of 70 to 160 nm and a thickness of ≈2.5 nm (Figure 3b–e). In another work, typical 2D polymer crystals were successfully synthesized through a two‐step pre‐crystallization/photo‐polymerization strategy including the pre‐crystallization of photoreactive monomer into a layered structure followed by a photo‐polymerization step.^[^ ^27^ ^]^ And the subsequent solvent‐induced delamination step (through heating at 150 °C in 1‐methyl‐2‐pyrrolidone for 3 days) successfully resulted in the formation of a high percentage of long‐ordered and mono‐layer nanosheets, and the atomic force microscope (AFM) images showed that the exfoliated single‐layer nanosheet had a uniform thickness of 2.5 nm. Agrawal's group also demonstrated a facile ambient‐pressure‐synthesis strategy to prepare highly crystalline layered structure poly (triazine imide) (PTI) and the subsequent simple exfoliation (based on stirring in anhydrous dimethylacetamide at 100 °C for 2 days) led to a large amount of crystalline single‐layer nanosheets with a mean thickness of 0.77 nm (Figure 3f–j).^[^ ^28^ ^]^

So far, most of the ultrathin 2D polymer nanosheets are synthesized by simple sonication‐ or heat‐assisted LPE exfoliation of bulk polymers with a layered structure. However, this strategy is difficult to achieve ultrathin nanosheets with high yields. In order to address this issue, Zhang's group developed a protonation‐assisted LPE method to improve the exfoliation yield of various N‐containing 2D polymer nanosheets from only 2–15% in pure water to 41–56% in 12 m HCl.^[^ ^29^ ^]^ The achieved ultrathin nanosheets had an average thickness of less than 5 nm, showing good dispersion in aqueous solutions. The high yield could be attributed to the fact that the presence of concentrated HCl would lead to the protonation of the N‐containing sites within the frameworks, which can produce strong electrostatic repulsion effect between adjacent layers and form hydrogen bonds with water molecules so that the neighboring layers within the bulk polymer could be easily exfoliated into ultrathin nanosheets.

In addition to the LPE process, chemical exfoliation is another significant exfoliation technique.^[^ ^30^ ^]^ For example, Banerjee et al. prepared anthracene‐based 2D COFs and chemically exfoliated the bulk COFs into 2D polymer nanosheets by using a simple cycloaddition reaction.^[^ ^31^ ^]^ It is a commonly known fact that the 9‐ and 10‐positions of anthracene units are sensitive toward [4 + 2] reaction. By using this character, the researchers introduced N‐hexylmaleimide into the anthracene moieties of the COF layers to initiate Diels–Alder reaction, which could disturb the *π*–*π* stacking interaction and the planarity of the COF layers. As a result, the bulk COF could be easily exfoliated in organic solvents, yielding high‐quality 2D polymer nanosheets without the need of sonication. Another typical example was reported by Fan et al.^[^ ^32^ ^]^ They found that layered CTF‐1 could be quickly and reversibly intercalated by using H_2_SO_4_ or H_3_PO_4_ to form CTF‐1 intercalated compounds, which could be easily reacted with nitrenium ions and finally exfoliated into ultrathin nanosheets with a thickness of 1.2–1.9 nm. However, in such strong acid media, the crystalline CTF‐1 structure was easily destroyed, thus leading to the formation of amorphous composition. More recently, Zheng et al. developed an azobenzene‐assisted chemical exfoliation strategy to obtain large, few‐layered nanosheets from bulk 2D COFs (Figure 3k).^[^ ^33^ ^]^ It is well known that the azo‐based molecules could transfer from the *trans‐* to *cis‐*isomer through the irradiation of UV light. Based on this photo‐isomerization phenomenon, the researchers introduced the azobenzene group into COF structures to break the interlayer interactions and tune the interlayer spacing so that COFs were successfully exfoliated into large‐scale COF nanosheets with a thickness of only 1.8 nm through simple UV radiation.

Apart from LPE and chemical exfoliation, mechanical exfoliation, relying on the external mechanical force to exfoliate bulk polymers into nanosheets, is also an effective and easy scale‐up strategy to obtain single‐ or few‐layer 2D polymer nanosheets. For example, we recently employed microcleavage exfoliation to exfoliate large‐size layered CTF crystals to obtain large‐area ultrathin nanosheets.^[^ ^34^ ^]^ However, this strategy usually suffers from the drawback of low yields. Mechanical grinding with a mortar is more efficient than the micromechanical cleavage.^[^ ^35^ ^]^ Benerjee's group synthesized a variety of 2D COF nanosheets by mechanically grinding bulk *β*‐ketoenamine‐linked COFs in a mortar (Figure 3l).^[^ ^36^ ^]^ The obtained COF nanosheets showed a lateral size of several micrometers and a thickness distribution ranging from 3 to 10 nm. In the view point of large‐scale production, utilizing a ball mill instead of a manual mortar and pestle may make mechanical grinding more automated and reproducible. In this regard, Wang et al. used a ball‐milling equipment to manufacture 2D polymer nanosheets without additional exfoliating agents from COFs as starting material.^[^ ^37^ ^]^ The resultant nanosheets showed ultrathin, transparent, and slightly wrinkled structures with a thickness of 3 to 5 nm.

Besides, some special methods have been reported to exfoliate bulk 2D polymers into nanosheets. For example, Liu's group found that the thermal oxidation etching approach could effectively exfoliate bulk g‐C_3_N_4_ into highly anisotropic 2D‐nanosheets.^[^ ^38^ ^]^ The inherent mechanism of this strategy was that the hydrogen bond‐linked cohered strands within the layer were susceptible to the oxidation process in air and would be gradually oxidized away from the bulk g‐C_3_N_4_, as a result of which fluffy 2D nanosheets could be successfully achieved. This solvent‐free and low‐cost method was thought to be very beneficial for the scale‐up production of g‐C_3_N_4_ nanosheets.

### 2.2 Bottom–Up Methods

The bottom–up technique is the one of the most common method for preparing regular molecular nanostructures by using covalent bonds to connect monomer molecules or structural components. It entails the efficient dispersion, rearrangement, and assembly of the building units in order to produce 2D polymer nanosheets via chemical processes.^[^ ^39^ ^]^ This technique could be used in the homogeneous solution,^[^ ^40^, ^41^, ^42^ ^]^ or at the interface, such as the liquid/solid,^[^ ^43^ ^]^ air/water,^[^ ^44^, ^45^, ^46^ ^]^ and liquid/liquid interfaces.^[^ ^47^ ^]^

**Figure 4:**
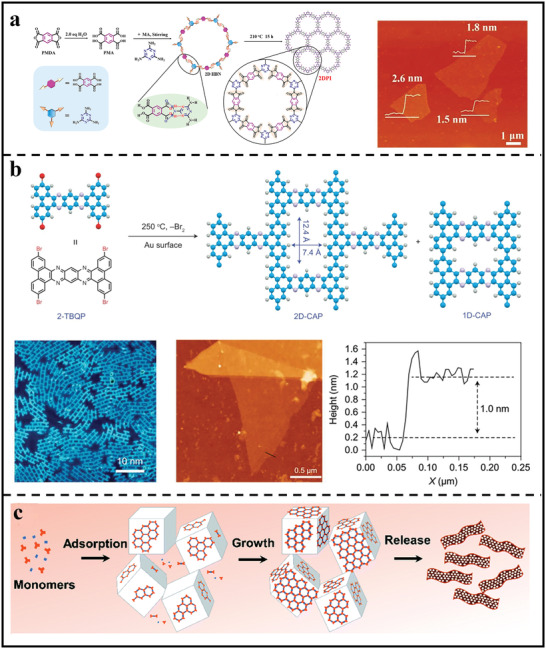
Syntheses of 2D polymer nanosheets through different bottom–up methods. a) Schematic illustration of the hydrogen bond‐directed 2D polymerization reaction to produce crystalline 2D polymer nanosheets. Reproduced with permission.^[^ ^41^ ^]^ Copyright 2019, American Chemical Society. b) Synthesis of CAP by the metal‐surface‐mediated polymerization and corresponding STM, AFM, and height images. Reproduced with permission.^[^ ^51^ ^]^ Copyright 2017, Springer Nature. c) Synthesis of imine‐linked COF nanosheets at the liquid/solid interface. Reproduced with permission.^[^ ^53^ ^]^ Copyright 2019, Royal Society of Chemistry.

In homogeneous solution synthesis, 2D polymer nanosheets are very prone to stacking into bulk materials due to various noncovalent interactions.^[^ ^48^, ^49^ ^]^ To synthesize 2D polymer nanosheets in one step, introducing repulsive interactions to disrupt the stacking is a very operative strategy. For example, Kim and coworkers reported a solution‐phase synthesis of readily transferable 2D polymers nanosheets.^[^ ^50^ ^]^ By taking advantage of the electrostatic repulsion of fully protonated spermines to keep each individual polymer nanosheet separated from one another during synthesis, single‐layer 2D polymer nanosheets could be successfully achieved in solution. In addition, Mitra et al. designed guanidinium‐based self‐exfoliated 2D polymer nanosheets.^[^ ^40^ ^]^ Benefiting from the introduction of cationic guanidinium moieties in the COF polymerization, electrostatic repulsive forces could protect nanosheets from stacking into robust bulk materials. As a result, ultrathin and crystalline 2D polymer nanosheets were successfully achieved, together with a thickness of 2–5 nm. Recently, our group also reported the efficient preparation of few‐layer crystalline 2D polyimide nanosheets via a two‐step synthetic strategy of hydrogen‐bond‐induced preorganization followed by imidization reaction (**Figure** **4**a).^[^ ^41^ ^]^ The achieved 2D polyimide nanosheets showed a crystalline characteristic with an average lateral size of ≈4 µm and a thickness of 1.5–2.6 nm. Moreover, the resultant nanosheets presented excellent dispersibility in ethanol (>2 months), demonstrating its excellent solution processability for membrane construction. Overall, this innovative solution‐phase method may enable the bulk manufacturing of easily transferrable 2D polymers in a practical and cost‐effective manner.

Different from homogenous solution synthesis, interfacial polymerization usually occurs at the interface with confined reaction zone, which makes it possible to achieve ultrathin nanosheets/nanofilms. Moreover, the nanosheets/film may be detached at any time during its growth process, allowing for precise control of thickness, crystallinity, and shape. For example, Loh et al.^[^ ^51^ ^]^ developed a crystalline 2D conjugated aromatic polymer (CAP) via metal‐surface‐mediated polymerization of the pre‐arranged tetrabromopolyaromatic monomer crystal on Au surface based on C–C coupling reaction (Figure 4b). The prepared 2D‐CAP exhibited an obvious layered stacking structure, from which ultrathin and micrometer‐sized 2D polymer sheets with a thickness of ≈1 nm can be readily obtained through simple mechanical exfoliation. Following liquid/solid interface reactions, Xu et al. carried out a Schiff base reaction on a highly oriented pyrolytic graphite surface at room temperature and obtained monolayer 2D polymer with pore sizes that varied from 1.7 to 3.5 nm depending on the size of the building units.^[^ ^52^ ^]^ To get a high yield of 2D polymer nanosheets, the interface should be increased. In this regard, Wang et al. described a salt‐template approach for producing few‐layered 2D polymer nanosheets (Figure 4c).^[^ ^53^ ^]^ A large number of NaCl particles were added into the reaction solution, resulting in abundant solid‐liquid interfaces. By controlling the reaction rate, the polymerization was largely limited to salt/solvent interface, which yielded large amounts of ultrathin nanosheets with thicknesses of a few nanometers.

**Figure 5:**
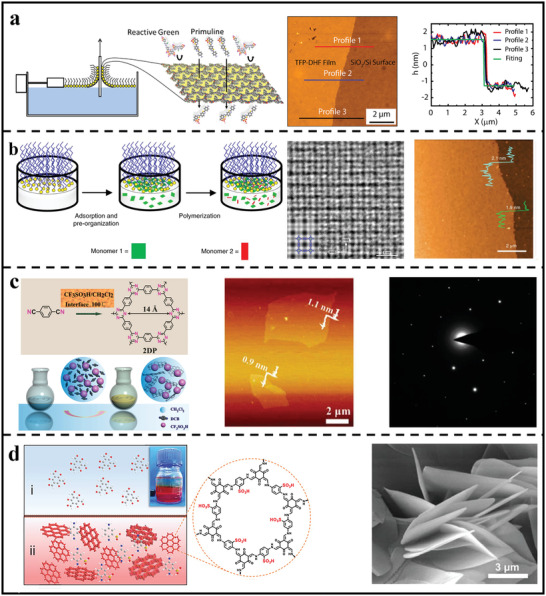
Syntheses of 2D polymer nanosheets through bottom–up methods at various interfaces. a) Synthetic procedure of few‐layer 2D polymer nanosheets at air/water interface through LB method and corresponding AFM image of the 2D polymer nanosheets. Reproduced with permission.^[^ ^54^ ^]^ Copyright 2019, American Chemical Society. b) Synthetic protocol of 2D polyimide nanofilm and corresponding HRTEM and AFM images of polyimide films. Reproduced with permission.^[^ ^55^ ^]^ Copyright 2019, Springer Nature. c) Schematic of dynamic liquid/liquid interfacial synthesis strategy toward crystalline 2D triazine nanosheets and corresponding AFM image and SAED pattern of the 2D triazine nanosheets. Reproduced with permission.^[^ ^60^ ^]^ Copyright 2017, American Chemical Society. d) Preparation of 2D polymer nanosheets at the liquid/liquid interface and corresponding SEM images of 2D polymer nanosheets. Reproduced with permission.^[^ ^61^ ^]^ Copyright 2020, Wiley‐VCH.

Similar to liquid/solid interface, air/water interface synthesis offers significant promise for 2D polymer nanosheets formation since extremely hydrophobic monomers may be constrained to the interface of a Langmuir monolayer before reaction.^[^ ^44^ ^]^ Over the past several years, great progress has been made on the preparation of 2D polymer nanosheets by using air/water interfacial synthesis. For example, in 2015, Schlüter et al. reported a self‐standing single‐layer 2D polymer film by photodimerization of anthracene‐based amphiphilic monomers at the air/water interface.^[^ ^46^ ^]^ By employing the air/water interface to constrain the precursor in a confined 2D space during polymerization, 2D polymer film with a finely ordered structure and ultrahigh pore density were successfully achieved on the water surface. Also, Lai's group efficiently synthesized 2D polymer film based on building units of 1,3,5‐triformylphloroglucinol and 9,9‐dihexylfluorene‐2,7‐diamine through the Langmuir–Blodgett (LB) method at air/water interface (**Figure** **5**a).^[^ ^54^ ^]^ The achieved 2D polymer film showed good freestanding ability and crystallinity. AFM measurements showed that the film thickness was about 2.9 nm. Recently, Feng and coworkers reported a surfactant‐monomer‐assisted gas/liquid interfacial synthesis (SMAIS) strategy to achieve crystalline 2D polymer films.^[^ ^55^ ^]^ As shown in Figure 5b, by utilizing the surfactant monolayers on the water surface to modulate the initial arrangement of monomers and subsequent 2D polymerization, ultrathin 2D polyimide, and polyamide nanofilms could be successfully achieved on the water surface. The achieved 2D polymers exhibited a high crystallinity and very large crystal domain size with a thickness of ≈2 nm. Based on this work, they further reported the preparation of porphyrin and triazine‐containing polyimine‐based 2D polymer films at the gas/liquid interface, which proved the universality of the SMAIS method for the syntheses of highly crystalline 2D polymer films.^[^ ^56^ ^]^

In addition to the liquid/solid and air/liquid interface, the liquid/liquid interface is another confined reaction zone, which is more versatile than the synthesis at the liquid/solid or air/liquid contact, making the construction of 2D polymer nanosheets easier.^[^ ^57^ ^]^ For example, Chung et al. constructed flexible and free‐standing 2D polymer films through confining the reaction of aldehyde and amine monomers at the interface between dichloromethane and water with the catalysis of CH_3_COOH.^[^ ^58^ ^]^ By adjusting the reaction time, the film thickness could be tuned from 5 to 300 nm. Apart from immiscible organic‐aqueous interface, Ma et al.^[^ ^59^ ^]^ reported the first example of growing 2D polymer nanosheets at the interface of two miscible organic solvents. They introduced a solvent interlayer as a buffer layer between two miscible solvents to control the monomer diffusion and reaction rate so that high‐quality polymer nanosheets were successfully achieved with a super‐large size (>200 µm^2^) and an ultrathin thickness of about 0.35 nm. The proposed method provided a facile and effective strategy to construct well‐structured 2D polymer nanosheets.

Apart from static liquid/liquid interface, our group recently developed a dynamic interfacial synthesis strategy to synthesize crystalline 2D polymer nanosheets (Figure 5c).^[^ ^60^ ^]^ In this method, CH_2_Cl_2_ was used to sufficiently dissolve the monomer and CF_3_SO_3_H was employed to catalyze the trimerization of nitrile groups. Due to the immiscibility of solvent and catalyst, lots of dynamic interfaces can be produced under vigorously stirring. It was believed that such abundant dynamic liquid/liquid interfaces were very favorable to control the reaction reversibility and van der Waals epitaxial effect, contributing to the construction of crystalline 2D polymer nanosheets. As a result, ultrathin single‐ or few‐layer 2D polymer nanosheets were successfully achieved with the thickness ranging from 0.9 to 3 nm.

More recently, Jiang et al. synthesized highly crystalline 2D polymer nanosheets by using a diffusion and solvent co‐mediated modulation approach under the oil‐water interfacial synthetic condition.^[^ ^61^ ^]^ As shown in Figure 5d, the synthetic system was composed of a top 1,3,5‐triformylphloroglucinol organic solution (dissolved in octanoic acid) and a bottom 2, 5‐diaminobenzenesulfonic acid (DABA) aqueous solution. In this case, an amorphous thin film was formed initially at the interface, which could slow down the diffusion rate of 1,3,5‐triformylphloroglucinol into DABA aqueous solution and lead to a slow nucleation and growth of 2D polymer nanosheets in the aqueous phase. Moreover, the strong affinity between the polymer nanosheets and water was conducive to prevent the nascent polymer nanosheets from aggregation and precipitation. As a result, high‐quality crystalline polymer nanosheets were successfully achieved in the aqueous phase with a thickness of 0.8 nm and a large lateral size up to 10 µm. It is worth noting that the resultant polymer nanosheets can be stable in water or other polar organic solvents for at least 20 months without any apparent precipitation, showing excellent dispersity.

To summarize, the primary drawback of “interfacial polymerization” techniques for producing 2D polymer nanosheets/nanofilms at interfaces is that they can only be produced at the interface contact region. Continuous regeneration of the phase interface is necessary to achieve a high yield of nanosheets/nanofilms. In addition, it might also be a difficulty to transfer freestanding 2D polymer nanofilms from the interface to other substrates.

## 3 Fabrication of 2D Polymer Nanosheets‐Based Membranes

Different approaches for assembling 2D polymer nanosheets into stacked structures with desired permeability and selectivity have been studied for laminar membranes depending on their structure and physicochemical characteristics. These preparation techniques usually have a substantial impact on the structure of the laminated membranes, including the interlayer spacing, thickness, and nanosheets arrangement, which could have an obvious effect on the membrane separation properties.^[^ ^62^, ^63^ ^]^ In most recent investigations, the focus of manufacturing methods is mainly on their capacity to accurately regulate the structure and performance of 2D membranes. Here, we classify the membranes into the following three types: 1) pure 2D polymer nanosheets‐based membranes, 2) MMMs, and 3) thin‐film nanocomposite (TFN) membranes and will discuss the preparation methods in each of them.

### 3.1 Pure 2D Polymer Nanosheets‐Based Membranes

The advent of 2D polymer nanosheets and their derivatives has provided the key to construct separation membranes that can overcome the “trade‐off” effect between permeability and selectivity. More channels for molecular transport are available thanks to the intrinsic pore volume of 2D polymer nanosheets‐based membranes. The functionality of 2D polymer nanosheets may be finely tuned by adding numerous functional sites on the organic building units by pre‐ or post‐synthetic modification. This enables for more precise regulation of pore size and hydrophilicity as well as the charge characteristic, which improves the selectivity and speeds up the separation process.

**Figure 6:**
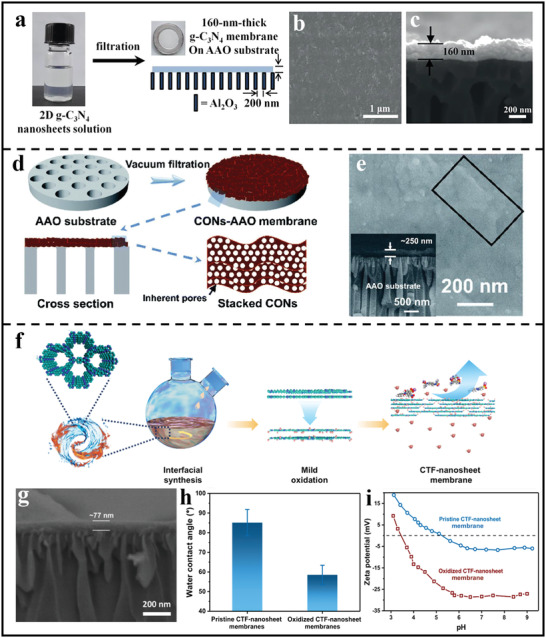
Composite membrane preparation based on 2D polymer nanosheets via vacuum‐assisted assembly. a) Fabrication process of g‐C_3_N_4_ membrane formed on AAO support. b) Surface and c) cross‐sectional SEM images of g‐C_3_N_4_ nanosheets‐assembled membrane supported on porous AAO substrate. (a–c) Reproduced with permission.^[^ ^65^ ^]^ Copyright 2020, American Chemical Society. d) Scheme of depositing imine‐linked 2D polymer nanosheets on AAO supports to prepare the composite membrane. e) SEM image of the membrane surface, inset is the cross‐sectional morphology of the composite membrane. (d,e) Reproduced with permission.^[^ ^53^ ^]^ Copyright 2019, Royal Society of Chemistry. f) Schematic diagram for preparing CTF‐nanosheets through dynamic liquid/liquid interface polymerization. g) Cross‐sectional SEM images of oxidized CTF nanosheets‐assembled membranes. h) Water contact angles and i) zeta potentials of the pristine and oxidized CTF nanosheets‐assembled membranes. (f–i) Reproduced with permission.^[^ ^66^ ^]^ Copyright 2020, American Chemical Society.

In order to assemble 2D polymer nanosheets into separation membranes, vacuum‐assisted filtration is a facile and effective strategy.^[^ ^64^ ^]^ As shown in **Figure** **6**a, 2D g‐C_3_N_4_ nanosheets were assembled into composite membranes through a simple vacuum‐assisted filtration strategy onto porous anodic aluminum oxide (AAO). It was clear from Figure 6b,c, that the assembled membrane exhibited a smooth and dense surface without any visible defects, together with an ultrathin thickness of 160 nm.^[^ ^65^ ^]^ Also, Wang et al. presented a salt‐template strategy to prepare few‐layered 2D polymer nanosheets and then deposited these nanosheets onto porous AAO substrates through vacuum‐assisted filtration assembly (Figure 6d).^[^ ^53^ ^]^ The as‐deposited film showed a complete and relatively smooth coverage on the support and the film thickness was about 250 nm (Figure 6e). It is believed that such dense membranes could be utilized in the separation of dye molecules. Furthermore, in order to endow the inherent hydrophobic CTF nanosheets with hydrophilic characteristics, Wang's group developed a mild oxidation strategy to prepare functionalized CTF nanosheets (Figure 6f).^[^ ^66^ ^]^ By utilizing dimethyl sulfoxide as a soft oxidant, simple ultrasonication would result in the formation of ultrathin and hydrophilic CTF nanosheets, which could stack onto porous substrates via vacuum‐assisted filtration to form separation membranes. The as‐synthesized membrane showed a smooth and dense surface and the thickness of active film was about 77 nm (Figure 6g). Moreover, thanks to the presence of oxygen‐containing groups, the oxidized membrane exhibited a better hydrophilicity and electronegativity when compared with the pristine CTF membrane (Figure 6h,i), which was expected to promote water transport and ion sieving. However, such composite membranes assembled form nanosheets usually possess some non‐selective defects and poor mechanical strength, which will lead to the unsatisfactory membrane performance.

**Figure 7:**
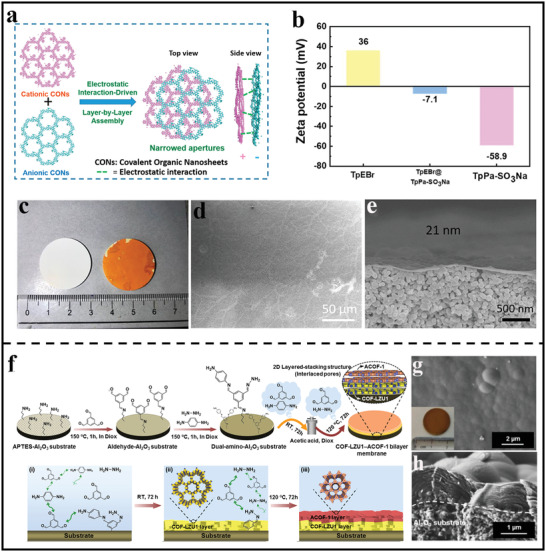
Preparation of continuous composite membrane based on 2D polymer nanofilms. a) Different stacking modes of 2D polymer thin films constructed. b) Surface charge properties of different nanosheets‐based membranes at pH 8.0. c) Optical images of the bare support and the nanosheets‐based membrane. d) SEM images of TpEBr@TpPa‐SO_3_Na nanosheets‐based membranes. e) Cross‐sectional SEM images of TpEBr@TpPa‐SO_3_Na nanosheets‐based membranes with 21 nm. (a–e) Reproduced with permission.^[^ ^67^ ^]^ Copyright 2020, American Chemical Society. f) Schematic diagram for synthesizing COF‐LZU1‐ACOF‐1 bilayer membrane. g) Surface and h) cross‐sectional image of the synthesized bilayer membranes. (f–h) Reproduced with permission.^[^ ^68^ ^]^ Copyright 2021, Elsevier.

Therefore, fabricating continuous and defect‐free 2D polymer membranes has become a charming pursuit. Interfacial synthesis provides a possible way to produce large‐area COF thin films and then the film could be directly deposited onto porous substrate through the layer‐by‐layer assembly (LBL) method. For example, Lai's group prepared a continuous and yellow imine‐bonded 2D COF‐based nanofilm at air/water interface via LB method and then deposited the nanofilm on AAO surface to form composite membranes.^[^ ^54^ ^]^ It is noting that the membrane thickness could be precisely tuned by controlling the nanofilm depositing layers, which is favorable to achieving optimal membrane performance. Afterward, Zhao's group also synthesized ultrathin 2D membranes via LbL assembly of two kinds of ionic COF nanosheets with different pore sizes and opposite charges (**Figure** **7**a).^[^ ^67^ ^]^ Benefiting from the staggered packing of ionic COF nanosheets with strong electrostatic interactions, the assembled membrane showed a narrowed pore size down to sub‐nanometer scale and a thickness of 21 nm (Figure 7b–e), which could be expected to achieve a selective gas separation through molecule sieving.

In situ solvothermal approach is another important method to obtain continuous defect‐free lamellar membranes. For example, Fan et al. fabricated COF‐COF bilayer composite membranes on a porous substrate through successively controlling the formation of imine‐linked COF‐LZU1 and azine‐linked ACOF‐1 layers through a temperature‐swing solvothermal strategy (Figure 7f).^[^ ^68^ ^]^ The resulting membrane showed a very dense surface combined with an obvious laminated structure composed of large amounts of nanosheets (Figure 7g,h). Besides, because of the in situ formed character, the obtained composite membranes possessed good interfacial adhesion between the support and the polymer layer, which was very suitable for the practical membrane applications.

### 3.2 Mixed‐Matrix Membranes

**Figure 8:**
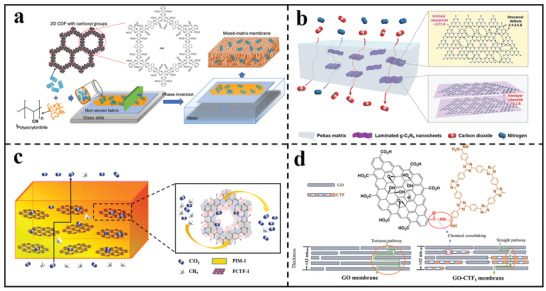
Preparation of MMMs through the incorporation of 2D polymer nanosheets into the polymeric matrix. a) Schematic illustration for the fabrication of a novel MMM by using PAN as the matrix and COF nanosheets as the fillers. Reproduced with permission.^[^ ^70^ ^]^ Copyright 2019, Elsevier. b) Synthesis protocols of a novel MMM through the introduction of g‐C_3_N_4_ nanosheets. Reproduced with permission.^[^ ^71^ ^]^ Copyright 2020, Elsevier. c) Synthesis of the PIM‐1/FCTF‐1 based MMM. Reproduced with permission.^[^ ^72^ ^]^ Copyright 2020, American Chemical Society. d) MMM preparation through the assembly from chemically grafted GO nanosheets and 2D CTF nanosheets. Reproduced with permission.^[^ ^73^ ^]^ Copyright 2019, American Chemical Society.

The objective of MMMs is to improve membrane permeance by mixing inorganic or inorganic–organic hybrid materials in the form of nanofillers with a polymer matrix. 2D polymer nanosheets are considered as promising candidates as porous fillers in polymeric matrix membranes to increase permeability by providing additional fast permeation channels, thus relieving the “trade‐off” limit between membrane selectivity and permeability.^[^ ^69^ ^]^ Moreover, the pure organic character of 2D polymer nanosheets made them more compatible with the polymer matrix than typical inorganic or inorganic–organic hybrid materials. Duong et al. reported a novel MMM through nonsolvent‐induced phase inversion by using PAN as the polymer matrix and carboxyl‐functionalized COF nanosheets as the nanofillers (**Figure** **8**a).^[^ ^70^ ^]^ It was found that increasing the concentration of COF nanosheets in the PAN casting solution would accelerate phase separation process, resulting in a more porous membrane with larger finger‐like macrovoids. Interestingly, with the addition of COF nanosheets, the resultant MMM surface exhibited a smaller effective pore size and a narrower pore size distribution. In another study, porous g‐C_3_N_4_ nanosheets were synthesized by thermal oxidation etching and incorporated into polyether block amide membrane (Figure 8b).^[^ ^71^ ^]^ The introduction of CO_2_‐philic g‐C_3_N_4_ nanosheets obviously improved the CO_2_ sorption capacity in the membrane, leading to a higher CO_2_/N_2_ sorption selectivity, which was expected to be beneficial for selective CO_2_/N_2_ membrane separation. Similarly, Jiang et al. prepared a novel MMM by solution casting method, which combined the advantages of 2D polymer nanosheets and polymers of intrinsic microporosity (PIMs) (Figure 8c).^[^ ^72^ ^]^ The addition of CO_2_‐philic perfluorinated CTF‐1 (FCTF‐1) nanosheets was employed to boost CO_2_ transport. Furthermore, the inherently pure organic character of FCTF‐1 was similar to that of the polymer matrix, allowing for superior polymer‐filler compatibility and the elimination of interfacial voids.

**Figure 9:**
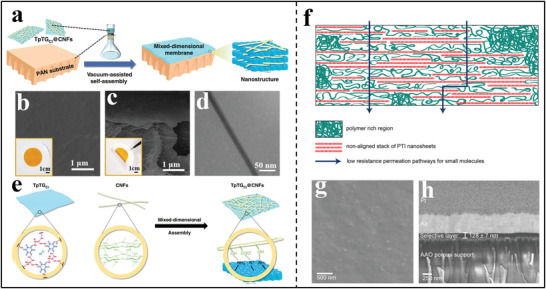
a) Schematic presentation for the fabrication of the mixed‐dimensional membrane through vacuum‐assisted self‐assembly. b) Surface SEM image of the TpTG_Cl_@CNFs‐5/PAN membrane, inset is a digital photo of the membrane. c) Cross‐sectional SEM image of the TpTG_Cl_@CNFs‐5/PAN membrane. d) Cross‐sectional TEM image of the TpTG_Cl_@CNFs‐5 membrane. e) Schematic illustration showing the assembly process and the interactions. (a–e) Reproduced with permission.^[^ ^74^ ^]^ Copyright 2020, Springer Nature. f) Diagram showing the selective layer of PTI membranes with m‐PBI chains as spacers. g) SEM image of the freestanding PTI/m‐PBI film. h) Cross‐sectional SEM image of the membrane. (f–h) Reproduced with permission.^[^ ^28^ ^]^ Copyright 2020, American Association for the Advancement of Science.

In addition to the traditional casting method, mixed self‐assembly is also another facile and effective strategy to construct MMMs. For example, Jiang's group synthesized two 2D materials: ‐NH_2_ functionalized CTF nanosheets and GO nanosheets.^[^ ^73^ ^]^ Then, the mixed solution containing hybrid nanosheets were assembled into a stacked membrane through vacuum‐assisted filtration assembly. Thanks to the strong electrostatic interaction between ‐NH_2_ and ‐COOH, the assembled membrane possessed a dense and robust structure (Figure 8d). In the GO‐CTF membranes, the CTF nanosheets provided a large number of in‐plane nanopores to shorten the molecular transport route while keeping the interlayer distance similar to that of GO membranes. In 2020, the same group used guanidinium‐based 2D polymer nanosheets and 1D cellulose nanofibers (CNFs) as building blocks to prepare a new MMM through mixed self‐assembly. (**Figure** **9**a–e).^[^ ^74^ ^]^ The negatively charged 1D CNFs were considered as the excellent candidates to reduce the pore size via covering onto the surface of COF nanosheets through sheltering effect. As a result, the sub‐nanometer pore size of 0.45–1.0 nm was successfully achieved, rendering the membranes precise molecular/ionic sieving properties. Moreover, the multiple interlamellar interactions between COF nanosheets and CNFs could endow the COF membranes with high structural stability. Recently, to overcome the nanopore blockage by nonaligned stacking of PTI nanosheets to some extent, Agrawal's group used m‐PBI (meta‐polybenzimidazole) chains as spacers between PTI nanosheets, which was accomplished via adding m‐PBI to the dispersion of PTI nanosheets followed by the vacuum‐assisted filtration assembly (Figure 9f–h).^[^ ^28^ ^]^ The obtained membrane showed a smooth, dense surface with an thickness of about 128 nm, and such ultrathin and defect‐free characteristics with the inherently sub‐nanopore structure of PTI were expected to hold promising potential for gas membrane separation.

### 3.3 Thin‐Film Nanocomposite Membranes

Generally speaking, TFN membranes, are fundamentally a modified form of thin‐film composite (TFC) membranes that blends the concepts of MMMs and TFC membranes.^[^ ^75^, ^76^, ^77^ ^]^ Over the past 30 years, polyamide‐based TFC membranes prepared through interfacial polymerization have been the most commercially successful membranes for water desalination. Traditional interfacial polymerization often produces an ultrathin skin layer over a hydrophobic substrate with low porosity, resulting in low water permeance. It is found that introducing porous fillers into the skin films is an effective strategy to improve the membrane permeability without a loss of salt rejection.

**Figure 10:**
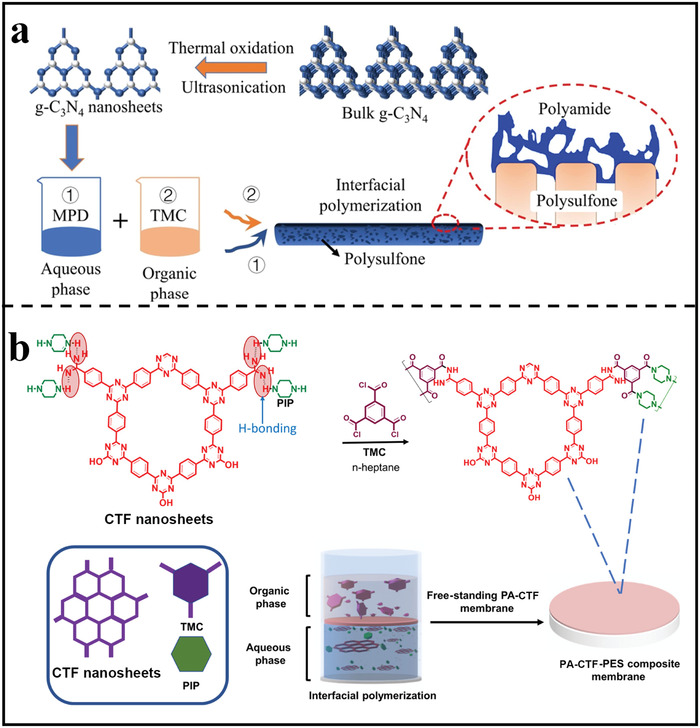
Fabrication of 2D polymer nanosheets‐incorporated TFN membranes. a) Schematic diagram for the fabrication of g‐C_3_N_4_ nanosheets‐incorporated TFN membranes. Reproduced with permission.^[^ ^79^ ^]^ Copyright 2021, Elsevier. b) Schematic diagram for fabricating CTF nanosheets‐incorporated TFN membrane. Reproduced with permission.^[^ ^80^ ^]^ Copyright 2020, American Chemical Society.

For example, Yuan et al. deposited 2D COF nanosheets on microfiltration membranes through vacuum‐assisted assembly prior to interfacial polymerization.^[^ ^78^ ^]^ The modified substrate was suitable for the uniform distribution of amine monomers due to the extremely porous structure and superhydrophilicity of COF nanosheets. The monomer storage capacity of the substrate may be increased via altering the loading amount of 2D COF nanosheets or increasing the concentration of amino monomers, resulting in faster self‐termination of interfacial polymerization and thus a thinner polyamide film. Furthermore, the extremely inherently porous nanosheets could provide additional water transport pathways so as to achieve a high permeance. In another work, Zhang et al.^[^ ^79^ ^]^ synthesized ultrathin and well‐dispersed g‐C_3_N_4_ nanosheets by two‐step thermal oxidation sintering and ultrasonic post‐treatment, and then the ultrathin g‐C_3_N_4_ nanosheets were incorporated into the polyamide separation layer through in situ interfacial polymerization process (**Figure** **10**a). Due to hydrophilic characteristics, g‐C_3_N_4_ nanosheets were well dispersed in the formed polyamide, which could obviously increase the membrane hydrophilicity and facilitate the transport of water molecule across the polyamide layer.

As for TFN membranes, improving the interfacial compatibility between nanofillers and polymer matrix is of great importance. It has been reported by Jiang's group that TFN membranes were fabricated by interfacial polymerization process using CTF nanosheets as active fillers (Figure 10b).^[^ ^80^ ^]^ The ‐NH_2_ reactive functional group in the peripheries of CTF nanosheets may react with the acyl chloride group, enhancing their affinity with the polyamide matrix and ensuring a high nanosheets loading quantity. The polymer nanosheets changed the physical and chemical architectures of membranes at the same time. Furthermore, by contributing hydroxyl and amine groups to the polyamide matrix, the chemical crosslinking of nanosheets with trimesoyl chloride during interfacial polymerization could greatly improve the interfacial compatibility. Similarly, Xu reported a novel TFN membrane comprising regular polymer nanosheets with high crystallinity.^[^ ^81^ ^]^ The 2D polymer nanosheets were first exfoliated from bulk COFs through sonication‐assisted LPE exfoliation and then dispersed in the amine containing aqueous solution. After interfacial polymerization, the exfoliated nanosheets were successfully incorporated within the polyamide network. Due the reactive ‐NH‐ sites on the polymer nanosheets with trimesoyl chloride, the obtained TFN membrane showed an excellent interfacial compatibility without the formation of any non‐selective defects.

## 4 Separation Applications of 2D Polymer Nanosheets‐Based Membranes

Benefiting from the inherently uniform and ordered pores, 2D polymer nanosheets are considered as promising candidates for constructing ultrafast and highly selective membranes. The size and function of the pores play important roles in determining the final separation performance of 2D polymer nanosheets‐based membranes. It is generally accepted that membrane separations are mainly based on the size exclusion mechanism (i.e., allow smaller molecules to transport across the membrane while reject molecules with the size larger the pore aperture). As for 2D polymer nanosheets, the pore size of polymer sheets (mostly in the range of 0.3–3 nm) would affect or even determine the average pore size of assembled 2D polymer membranes, thereby defining the final separation applications such as gas separation, reverse osmosis (RO), nanofiltration (NF), ultrafiltration (UF), and so on. Basically, there are mainly two kinds of strategies to control the pore size of 2D polymer nanosheets. The first method, from the view point of molecular design, is to change organic linker units with different geometries and lengths to determine the polygon shape (e.g., tetragonal, rhombic, trigonal, and hexagonal structures) and dimensions of 2D polymer structures so as to control the pore size. For example, when a trigonal linker is combined with a linear linker, tetragonal pores could be formed and the dimensions of the tetragonal pores are tunable by varying the length of the linear linkers and the size of the trigonal linker. By utilizing this method, Banerjee's group^[^ ^82^ ^]^ developed a series of COF‐based membranes with the pore size ranging from 1.4 to 2.6 nm through selecting building units with different lengths. The other strategy of adjusting pore size is to introduce large side groups or functional groups within 2D polymer structure through pre/post‐functionalization, and this strategy greatly expands the adjustable range of pore sizes beyond the limited number of organic linkers available for 2D polymer synthesis. For example, Huang et al.^[^ ^83^ ^]^ synthesized a 2D carboxyl‐functionalized COF membrane via post‐modification based on ring‐open reaction of succinic anhydride with hydroxyl‐containing COF. Through this post‐modification, the pore size of the membrane was obviously constricted, which was helpful to improve ionic/molecular selectivity based on size sieving mechanism.

In addition to pore size, the interactions between the transported molecules and 2D polymer membranes, including charge properties, hydrophilicity/hydrophobicity, and so on, also play important roles in determining the membrane perm‐selectivity, especially in aqueous and solvent‐based separations. Membranes with the same charged characteristics as the solute tend to have relatively high rejections based on the Donnan effect (also known as electrostatic repulsion effect) between the membrane surface and the solutes. For example, it was found that positively charged membrane assembled from cationic polymer nanosheets showed a higher rejection toward cationic dyes than those of anionic dyes.^[^ ^84^ ^]^ As for hydrophilicity/hydrophobicity, a computational study showed that 2D COF‐based membranes with hydrophilic functional groups (e.g., ‐NH_2_, ‐OH, and ‐COOH) exhibited a higher pure water flux than their hydrophobic counterparts of similar pore size for water desalination. This was mainly due to the preferential interaction between water and hydrophilic groups, which could lead to a faster transport of water molecules across the membrane.^[^ ^85^ ^]^ Besides, it is worthy to note that the interaction between membrane and gas molecules also have an obvious effect on the final separation performance. As for CO_2_ capture and separation, when the 2D polymer nanosheets contains enriched basic N atoms, the CO_2_ permeance and corresponding selectivity of CO_2_ with other gases could be apparently improved due to the strong affinity between acidic CO_2_ and basic N atoms.^[^ ^86^ ^]^ In the following section, we will concentrate on the diverse separation applications of 2D nanosheet‐based membranes, followed by a brief discussion on how 2D polymer nanosheets could improve the separation performance of pure 2D polymer nanosheets‐based membranes, MMMs, and TFN membranes.

### 4.1 Gas Separation

Traditional amorphous polymer membranes have a disorganized and uneven pore size, making it difficult to achieve a high perm‐selectivity that exceeds the present Robeson up bound.^[^ ^87^ ^]^ However, ultrafast and extremely selective molecular sieving may be achieved using 2D polymer nanosheet‐based membranes with numerous and well‐ordered in‐plane pores. Zhong's group has conducted a computational study to explore the gas separation properties of 2D CTF‐based ultrathin membranes.^[^ ^88^ ^]^ The results showed that ultrathin 2D polymer nanosheets‐based membranes have many advantages for gas separation: 1) the narrow interlayer galleries between stacked nanosheets may have a “gate‐closing” impact on selective molecular sieving; 2) adjusting the nanosheets stacking modes could generate a favorable energy microenvironment that might be regarded a useful regulatory and control technique for preparing ultrathin membranes with high gas permeability and selectivity.

**Figure 11:**
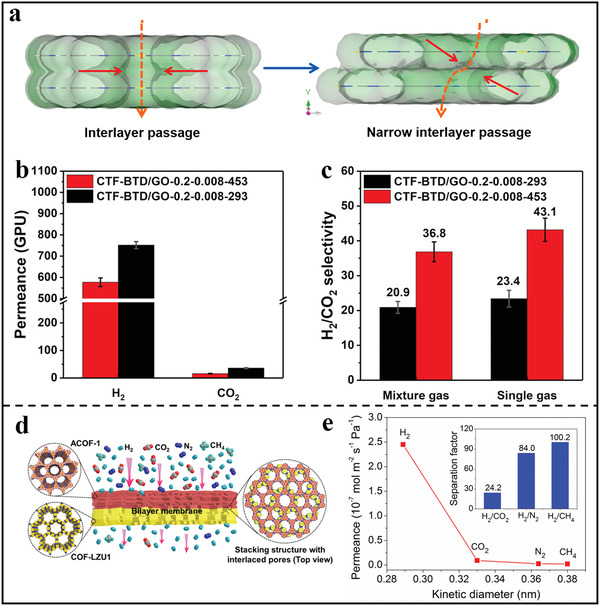
2D polymer nanosheets‐based membranes for gas separation. a) Scheme of the difference of the interlayer passage. b) Gas permeance of CTF‐BTD/GO membranes tested with H_2_/CO_2_ (v/v = 1/1) mixed feed gas. c) H_2_/CO_2_ mixed gas separation factor and single gas ideal selectivity for CTF‐BTD/GO membranes. (a–c) Reproduced with permission.^[^ ^90^ ^]^ Copyright 2018, American Chemical Society. d) Structures of COF‐LZU1/ACOF1 bilayer membranes. e) Single gas permeance of the COF‐LZU1/ACOF1 bilayer membrane, inset shows the mixed gas separation factor. (d–e) Reproduced with permission.^[^ ^68^ ^]^ Copyright 2021, Elsevier.

The pioneering experiment work of 2D materials applied in gas separation was described by Park's group.^[^ ^89^ ^]^ They found that the narrow interlayer channels in the GO membranes could be utilized as gas permeation and separation channels. Compared with inorganic 2D materials, the porous 2D polymer nanosheets feature a large number of pores, which can significantly improve gas permeability. In 2017, Li et al. fabricated high‐quality 2D laminated membrane on a porous ceramic support via drop‐coating.^[^ ^18^ ^]^ Benefiting from the inherent perforations on the 2D COF‐1 nanosheets, the nanosheets‐based membrane showed an excellent H_2_ permeance of 10^6^ mol m^−2^Pa^−1^s^−1^. However, it should be pointed that the gas selectivity was relatively low due to the loose stacking structure. In order to improve membrane selectivity, Zhao et al. used 2,1,3‐benzothiadiazole‐4,7‐benzenedicarbonitrile (BTD) as the building units to synthesize CTF and then fabricated ultrathin CTF‐BTD/GO membranes via the restacking of the nanosheets with the help of heating‐driven filtration assembly process.^[^ ^90^ ^]^ Such strategy could optimize the stacking arrangement of nanosheets without sacrificing thickness control, enabling the membrane defect‐free and ultrathin features along with the reduced pore size down to sub‐nanometer scale (**Figure** **11**a). The resultant membrane exhibited an excellent H_2_ permeance and H_2_/CO_2_ selectivity (Figure 11b,c), which were superior to those of the membranes synthesized under room temperature.

**Figure 12:**
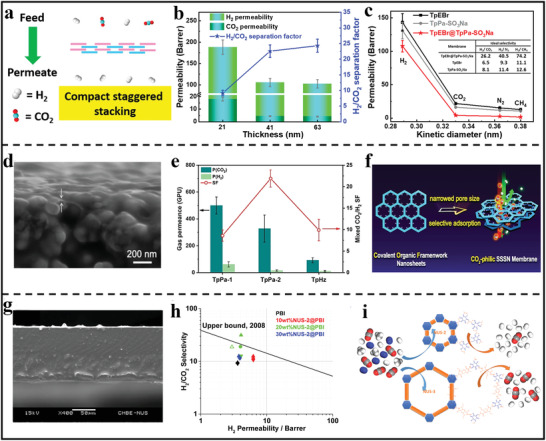
2D polymer nanosheets‐based membranes for gas separation. a) The scheme of H_2_/CO_2_ transport behavior through the assembled membranes formed by compact staggered stacking of ionic nanosheets. b) The relationship of thickness of membranes and H_2_/CO_2_ separation performance. c) Single component gas permeation of different nanosheets‐based membranes. (a–c) Reproduced with permission.^[^ ^67^ ^]^ Copyright 2020, American Chemical Society. d) Cross‐sectional SEM image of 2D TpPa‐2 membrane. e) The binary CO_2_/H_2_ separation performances of three kinds of 2D polymer membranes f) Illustration of gas permeation behaviors. (d–f) Reproduced with permission.^[^ ^91^ ^]^ Copyright 2021, Wiley‐VCH. g) Cross‐sectional SEM image of MMMs. h) Mixed gas permeation properties of MMMs. i) Illustration of gas selectivity of the obtained MMMs. (g–i) Reproduced with permission.^[^ ^92^ ^]^ Copyright 2016, American Chemical Society.

Taking into account the staggered stacking mode, the Caro's group prepared a novel COF‐COF bilayer membrane by depositing another 2D COF layer on top of first 2D COF layer through in situ solvothermal synthesis, which featured an interlaced pore at the interface of two COF layers (Figure 11d).^[^ ^68^ ^]^ Benefiting from the formation of interlaced pore networks, the pore size of bilayer membranes reduced down to sub‐nanometer scale, which could be used in precise gas sieving. As exhibited in Figure 11e, the obtained membrane showed a higher H_2_ permeance of 2.45 × 10^−7^ mol m^−2^s^−1^Pa^−1^ to those of the other gases. Furthermore, when the temperature increased from 298 to 393 K, the permeance of H_2_ increased from 1.9 × 10^−7^ to 5.0 × 10^−7^ mol m^−2^s^−1^Pa^−1^, which also maintained high thermal and operational stability. In addition to in situ solvothermal, LBL is another facile and effective strategy to construct dense membranes with compact staggered stacking structure. For example, Zhao's group reported the fabrication of ultrathin 2D membranes for gas separation through LbL assembly of two kinds of ionic polymer nanosheets (**Figure** **12**a).^[^ ^67^ ^]^ As demonstrated in Figure 12b, as the membrane thickness increased, the permeability dropped and the separation factor increased, benefiting from the formation of the denser membrane with a compact staggered stacking structures. Under optimal fabrication condition, the obtained membrane with a thickness of 41 nm had an attractive H_2_ permeance of 105.2 Barrer. As for the molecular sieving behavior, it can be observed from Figure 12c that there was a sharp cut‐off of the permeance between H_2_ and other larger gases, implying a clear molecular sieving property.

More recently, Yang's group designed and synthesized three kinds of *β*‐ketoenamine‐based 2D polymer nanosheets with different pore sizes and then assembled the ultrathin nanosheets into the dense film on *α*‐Al_2_O_3_ substrate through a hot‐drop coating method (Figure 12d).^[^ ^91^ ^]^ Benefiting from the staggered stacking pattern of polymer nanosheets, the effective aperture of the assembled membrane was greatly reduced down to sub‐nanometer size, which was suitable for gas separation. As shown in Figure 12e, the best TpPa‐2 membranes with a medium pore size showed a high CO_2_ permeance of 328 GPU and an excellent CO_2_/H_2_ separation factor of 22 and at 298 K, reaching the target for commercial syngas separations. This excellent separation properties mainly attributed to the narrowed pore sizes and CO_2_‐philic adsorption capacities originated from abundant CO_2_‐philic amine sites within 2D polymer nanosheets (Figure 12f). Besides, Agrawal's group^[^ ^28^ ^]^ demonstrated that the PTI nanosheets‐based membranes were extremely attractive for high‐temperature hydrogen sieving due to the inherent sub‐nanometer pore size of PTI nanosheets. They found that the PTI membranes with m‐PBI chains as spacers exhibited an excellent H_2_ permeance (900 to 1450 GPU) and corresponding H_2_/CO_2_, H_2_/N_2_, and H_2_/CH_4_ selectivities of 10, 48, and 63, respectively. Moreover, in order to evaluate the practical separation performance of PBI‐impregnated PTI membranes, they tested the membrane using a 50:50 mixture feed of H_2_ and N_2_ at 250 °C. The experimental test result showed that the H_2_ permeance and the H_2_/N_2_ separation factors agreed well with that of the single‐gas test, holding promising potential in various applications including regulating the H/N ratio in ammonia synthesis and H_2_ purification after the water‐gas shift reaction.

To date, there are only a very few reports of continuous 2D polymer‐based membranes as sieving membranes for gas separations. This may be due to fact that the pore size of most 2D polymer (>1 nm) is much larger than the kinetic diameters of gases. Moreover, the weak interlayer interaction between 2D polymer nanosheets hinders the fabrication of defect‐free membranes. Therefore, the addition of 2D polymer nanosheets into polymeric matrix has become a facile and effective strategy and the resultant composite membranes are known as MMMs. For example, Zhao's group synthesized two water‐stable 2D polymer nanosheets and then mixed them with commercial polymers poly(ether imide) or polybenzimidazole into MMMs (Figure 12g).^[^ ^92^ ^]^ The resultant membranes exhibited highly homogeneous textures resulting from the good compatibility between polymer nanosheets and the polymer matrix. Benefiting from the selective gas sorption properties of the porous polymer nanosheets (Figure 12i), the optimized MMM exhibited an increased gas permeability, showing an excellent H_2_/CO_2_ perm‐selectivity that exceeded the 2008 Robeson upper bound (Figure 12h). However, the permeance (4.08 ± 0.03 Barrer) was still too low for commercial applications. Recently, Duan et al. fabricated the MMM through spin‐coating 2D polymer nanosheets‐containing polyether‐block‐amide (PEBA) solution on PVDF supports.^[^ ^93^ ^]^ On account of the functional amine groups and uniform pore structure, TpPa‐1 polymer nanosheets had a good CO_2_/N_2_ adsorption selectivity. As a result, the PEBA‐based MMMs with only 1 wt% polymer nanosheets exhibited a high CO_2_/N_2_ selectivity (≈72), showing great potential for large‐scale selective CO_2_ removal from N_2_.

### 4.2 Water Purification

2D polymer nanosheets‐based membranes have uniform and ordered pore size, tunable hydrophilicity, and good mechanical robustness, holding great promise for water purification. Consequently, 2D polymer nanosheets‐based membranes have been developed by many researchers for a wide range of water purification applications.^[^ ^94^, ^95^, ^96^, ^97^ ^]^ We will introduce them from the following two aspects: wastewater treatment and water desalination. In terms of wastewater treatment, the removal of molecules such as dyes will be mainly discussed.

#### 4.2.1 Wastewater Treatment

Water contamination is a significant global issue that has ramifications for both the environment and human health. The long‐term management of water resources necessitates the development of modern wastewater treatment technology. As a result, developing effective, low‐cost wastewater treatment and reuse technologies has become a priority. Membranes in water and wastewater administration are one promising method that has seen success in numerous investigations. The rapid advancement of membrane science research demonstrates its rising potential.

**Figure 13:**
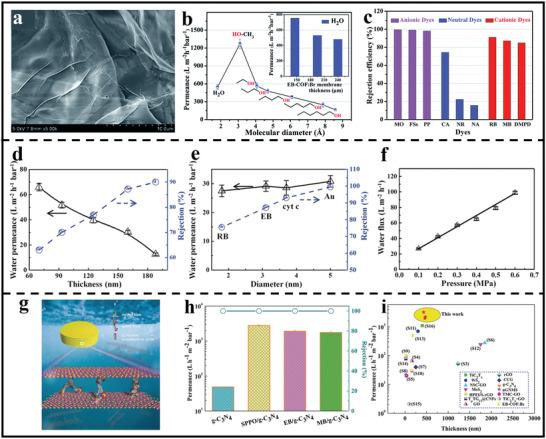
2D polymer nanosheets‐based membranes for wastewater treatment. a) Cross‐sectional SEM image of 2D polymer nanosheets‐based membrane. b) Pure solvent permeance versus different molecular diameter. c) The rejection efficiency of the membrane for different dyes. (a–c) Reproduced with permission.^[^ ^84^ ^]^ Copyright 2018, Royal Society of Chemistry. d) The water permeances of g‐C_3_N_4_ membranes with different thickness. e) Separation performance of g‐C_3_N_4_ based membranes for different molecules. f) Water flux of g‐C_3_N_4_ membranes under different pressure. (d–f) Reproduced with permission.^[^ ^65^ ^]^ Copyright 2017, Wiley‐VCH. g) Schematic diagram of separation process of g‐C_3_N_4_ based membrane. h) Permeances and rejections of different membrane tested with MB solutions. i) Permeance comparisons of g‐C_3_N_4_ based membrane with the other 2D membranes reported in the literature. (g–i) Reproduced with permission.^[^ ^98^ ^]^ Copyright 2019, Wiley‐VCH.

In the past several years, great progress has been made on 2D polymer nanosheets‐based membranes applied in wastewater treatment. For example, Zhang et al. prepared positively charged 2D polymer membranes through a vacuum‐assisted filtration assembly of cationic COF nanosheets.^[^ ^84^ ^]^ The resulting membrane had a rapid solvent penetration though optimizing the film thickness. More importantly, thanks to the abundant positive charge sites within pore walls, the assembled membrane demonstrated precise molecule sieving property for dye molecules with different charges. As shown in **Figure** **13**a–c, the composite membrane could efficiently reject ≈98% of anionic dye molecules, while maintaining an excellent solvent permeance. Recently, Zheng et al. also reported the successful preparation of ultrathin 2D membranes from azobenzene‐containing 2D polymer nanosheets.^[^ ^33^ ^]^ Because of the inherent porous character, the as‐assembled membrane exhibited a higher water flux (596 L m^−2^h^−1^bar^−1^) than GO‐based membranes (4.3 L m^−2^h^−1^bar^−1^), while maintaining a high rejection of dye molecules. In addition to 2D COF nanosheets, the inherent porous g‐C_3_N_4_ nanosheets could also be utilized to construct membranes for wastewater treatment. Caro et al. has prepared g‐C_3_N_4_ membrane through the assembly of partially exfoliated g‐C_3_N_4_ nanosheets. Attributing from the presence of transport pathways including artificial nanopores (sizes between 1.5 and 3 nm) and the interlayer passage between the nanosheets (produced by unstripped fragments), the optimized 160‐nm‐thick g‐C_3_N_4_ membrane exhibited a good water permeance of 29 L m^−2^h^−1^bar^−1^, together with rejection rates of 75.5%, 87.2%, 93.1%, and 99.5% for rhodamine B, Evans Blue, cytochrome c, and Au nanoparticles (diameter 5 nm), respectively (Figure 13d,e). Moreover, it was found that water flux also exhibited a linear increase with the increased pressure and with constant rejection (Figure 13f), indicating the enough rigidness and mechanical properties of nanochannels within g‐C_3_N_4_. Besides, molecular dynamics simulations demonstrated that water transport through the g‐C_3_N_4_ nanosheets with ultralow friction. In order to further improve the water permeance of g‐C_3_N_4_ membranes, Ran and Xu et al.^[^ ^98^ ^]^ intercalated molecules with ‐SO_3_H and benzene moieties between layers to break up the tightly stacking structure of g‐C_3_N_4_ laminates and enlarge interlayer channel (Figure 13g). As a result, the modified g‐C_3_N_4_ membranes possessed an extraordinary water permeance of 8867 L h^−1^m^−2^bar^−1^, two orders magnitude higher than that of the pristine membrane, without sacrificing the separation efficiency (100% rejection toward methyl blue) (Figure 13h,i). Furthermore, the introduction of SO_3_H sites firmly anchored N atoms within g‐C_3_N_4_ through acid–base interactions, enabling the excellent stability of the nanochannels of g‐C_3_N_4_ based membranes in harsh environments.

Apart from the assembly from single 2D polymer nanosheets, mixed assembly could also be used to construct high‐performance separation membranes, which combines the advantage of different materials. For example, hybrid GO/COF‐1 nanocomposite membranes have been synthesized through in situ growth of COF‐1 on the surface of GO nanosheets followed by mixed assembly.^[^ ^99^ ^]^ The prepared membranes exhibited good water permeability (310 L m^−2^h^−1^Mpa^−1^), together with a high rejection for water‐soluble dyes (>99%).

#### 4.2.2 Water Desalination

Under the urgent demand to improve desalination performance, salt ion‐sieving membranes have attracted increasing interests over the past few years. The basic principle of ion‐sieving membranes is based on a combined effect of size exclusion and Donnan exclusion mechanism.^[^ ^100^, ^101^ ^]^ In 2017, Loh et al. reported a novel 2D CAP through the C–C coupling reaction between tetrabromopolyaromatic monomers.^[^ ^51^ ^]^ Because of the highly ordered sub‐nanometer pores, the ultrathin 2D CAP membrane showed promising potential for water desalination. Thus, they conducted molecular dynamics simulations to analyze the desalination properties of 2D‐CAP membranes. The simulation results showed that the bilayer CAP membrane could reject almost all the salt ions (100%), together with a high water permeance (1172 L m^−2^h^−1^bar^−1^), which is three orders of magnitude higher than the commercially available reverse osmosis membranes.^[^ ^102^ ^]^ Similar simulation results were also reported by Grossman's group and Jiang’ team.^[^ ^85^, ^103^ ^]^ All these results demonstrated 2D polymer nanosheets could be considered as the promising candidates for membrane‐based separation in water desalination.

**Figure 14:**
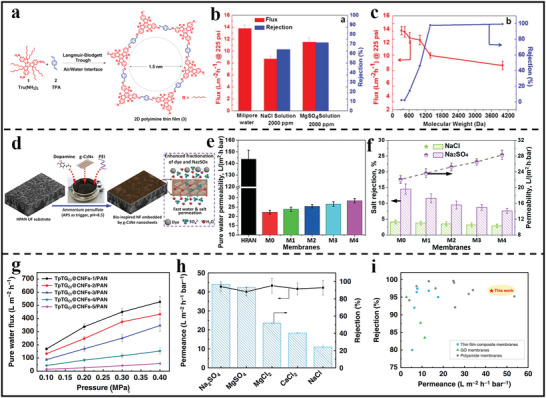
2D polymer nanosheets‐based membranes for water desalination. a) Scheme of the fabrication for 2D polymer‐based thin film through LB method at air/water interface. b) Pure‐ and salt‐water flux of the 2D polymer‐based thin film. c) Flux and rejection of the 2D polymer‐based thin film tested using dyes with different molecular weight. (a–c) Reproduced with permission.^[^ ^104^ ^]^ Copyright 2018, American Chemical Society. d) Schematic of co‐deposition of dopamine/PEI and g‐C_3_N_4_ nanosheets triggered by APS on HPAN substrate. e) Pure water permeability and f) salt‐water filtration performance of the bio‐inspired membranes, respectively. (d–f) Reproduced with permission.^[^ ^105^ ^]^ Copyright 2019, Royal Society of Chemistry. g) Pure water flux of the TpTGCl@CNFs‐X/PAN membranes at different pressure. h) Permeance and rejection of the TpTGCl@CNFs‐4/PAN membrane for the nanofiltration of different salt solutions. i) Comparison of the permeance and rejection of the TpTGCl@CNFs‐4/PAN membrane with the representative membranes reported in the literatures. (g–i) Reproduced with permission.^[^ ^74^ ^]^ Copyright 2020, Springer Nature.

In 2018, Lai's group demonstrated an innovative and simple synthesis of 2D polymer‐based film with pore size around 1.5 nm using a combined strategy of LB method and self‐assembly (**Figure** **14**a).^[^ ^104^ ^]^ Thanks to the uniform and ordered pore, the obtained membrane exhibited good salt rejections for both monovalent and divalent salts (64.27% and 71.34% for NaCl and MgSO_4_ solutions, respectively). Meanwhile, the membrane showed a high pure water flux of around 13.8 L m^−2^h^−1^ (Figure 14b). It was clear from Figure 14c that the cut‐off molecular size was ≈1.3 nm, close to the predicted membrane pore size. In another study, Zhou et al.^[^ ^105^ ^]^ developed high‐flux NF membranes through bio‐inspired co‐deposition of hydrophilic g‐C_3_N_4_ nanosheets with polydopamine (PDA)/polyethylenimine (PEI) triggered by ammonium persulfate (APS) onto porous hydrolyzed PAN substrate (Figure 14d). The introduction of g‐C_3_N_4_ nanosheets produced additional nanochannels in the PDA/PEI layer to facilitate water molecule transport, as a result of which a high water permeance was successfully achieved (28.4 ± 1.2 L m^−2^h^−1^bar^−1^). However, due to the relatively loose structure of co‐deposition layer, the salt rejection was extremely low (2.9% for NaCl; 7.6% for Na_2_SO_4_) (Figure 14e,f). In addition, tuning the staking mode is also another effective strategy to construct precisely sieving desalination membrane. For example, Ma et al. changed the AA stacking mode of 2D COF layers to AB stacking mode in order to lower the pore size from >1 nm to sub‐nm. The resulting AB stacking COF membrane, which was made up of highly‐ordered nanosheets, had a narrow pore size (0.6 nm) and an excellent ion sieving performance.^[^ ^106^ ^]^

The desalination applications discussed above are mainly focused on pure 2D polymer nanosheets‐based membranes. Although such membranes exhibited high water permeance due to their intrinsically porous characteristics, their salt rejection is relatively lower than commercially available polymeric membranes. Therefore, combining 2D polymer nanosheets and traditional polymeric membrane becomes a feasible method to construct TFN membranes with both high permeance and salt rejection. For example, Jiang's group developed CTF nanosheets‐based TFN membrane for water desalination.^[^ ^107^ ^]^ The porous and hydrophilic characteristic of CTF nanosheets contributed to facilitate water transport through the membrane. In addition, the reaction between CTF nanosheets and trimesoyl chloride formed strong covalent bonds, which is favorable to improve the interfacial compatibility and avoid the production of non‐selective defects. As a consequence, the modified TFN membrane produced a high flux of 23.8 L m^−2^h^−1^bar^−1^, which was 5 times higher than that of the unmodified polyamide membrane, together with a high Na_2_SO_4_ rejection of (>95%). In addition, between the polyamide layer and the PAN substrate, a PDA TpPa‐1 COF hybrid porous intermediate layer was inserted in a recent research.^[^ ^108^ ^]^ The membrane structural integrity and water flux showed an obvious improvement while the Na_2_SO_4_/MgSO_4_ rejection still remained outstanding.

In addition to TFN membranes, mixed assembled membrane could also have outstanding desalination performance. Recently, Jiang et al. proposed a concept of combining membrane materials with different dimensionality.^[^ ^74^ ^]^ The mixed assembly limited the pore size of COF membranes and increased their mechanical robustness. As a result, the developed MMM exhibited a high permeance of 42.8 L m^−2^h^−1^bar^−1^ and a good rejection of 96.8% for Na_2_SO_4_, which exceeded most of the membrane performance reported in the literatures (Figure 14g–i).

### 4.3 Organic Solvent Separation

**Figure 15:**
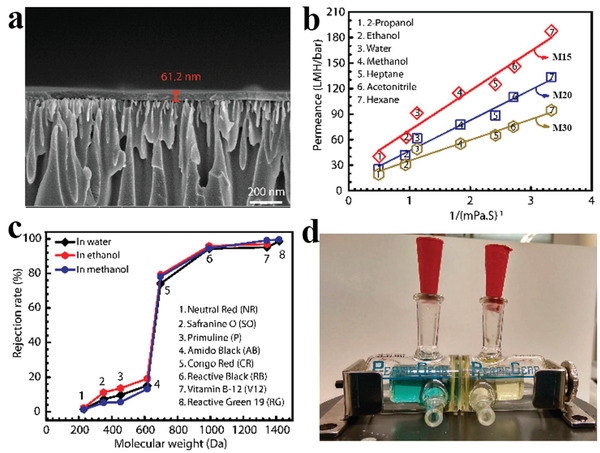
2D polymer nanofilms‐based composite membranes for organic solvent separation. a) Cross section SEM image of 2D COF nanofilms‐based membranes. b) Permeances of different solvents through the three 2D COF nanofilms‐based membranes. c) Rejection of various dyes. d) Image showing the separation of the mixture dyes of reactive green and primuline. Reproduced with permission.^[^ ^54^ ^]^ Copyright 2018, American Chemical Society.

With the growth of the pharmaceutical, catalytic, and petrochemical sectors, organic solvent separations are becoming increasingly essential.^[^ ^109^, ^110^, ^111^ ^]^ As a result, there is a pressing need to prepare new membranes to bridge the technical gap and improve membrane capacity in organic solvents. The fast development of 2D polymer materials opens up exciting possibilities for expanding membrane applications into organic liquids.^[^ ^84^, ^112^ ^]^ For example, Banerjee's group^[^ ^82^ ^]^ developed a liquid/liquid interfacial polymerization method to construct ultrathin freestanding COF membranes in 2017. The optimized 2D COF membranes exhibited outstanding permeance to acetonitrile at 339 L m^−2^h^−1^bar^−1^, together with a high dye rejection. The only drawback was that it took relatively long time (usually 72 h) to obtain the crystalline COF thin film. Afterward, Dichtel et al. found that Sc(OTf)_3_ could highly catalyze the formation of imine‐linked COF with excellent crystallinity in a short period at room temperature.^[^ ^113^ ^]^ Based on this discovery, they synthesized ultrathin imine‐based COF nanofilms through interfacial polymerization, with a tunable thickness from 2.5 nm to 100 µm.^[^ ^114^ ^]^ The free‐standing COF films could be transferred onto PSf supports, and the optimal composite membrane exhibited an enhanced rejection of Rhodamine WT which was up to 91%. In another study, a series of imine (–C═N–) linked porous crystalline 2D polymer nanosheets were synthesized with a salt‐mediated strategy, which could be assembled into composite membrane.^[^ ^53^ ^]^ Due to the tightly packed stacking of polymer nanosheets with thin thicknesses on the porous support membrane, the assembled membrane exhibited excellent sieving capacity for similarly sized dye molecules, while maintaining an ultrafast water permeation. In addition, Lai's group successfully synthesized composite membrane through LBL assembly of COF nanofilms on AAO porous support.^[^ ^54^ ^]^ The resultant 61‐nm‐thick membrane displayed excellent permeances for both polar and nonpolar organic solvents, which could be mainly attributed to the well‐defined ordered porous structures (**Figure** **15**a). Moreover, the membrane showed a steep molecular sieving property with a molecular weight cut‐off of about 900 Da (Figure 15b,c).

**Figure 16:**
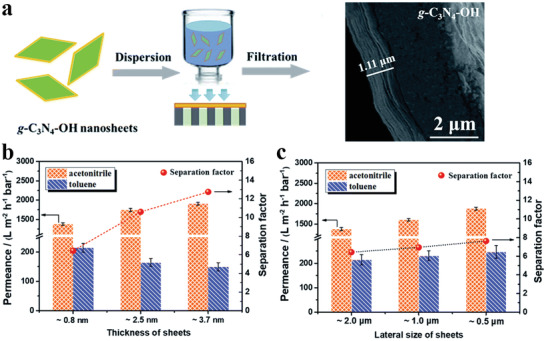
g‐C_3_N_4_‐OH nanosheets assembled membranes for water desalination. a) Schematic illustration of the preparation of g‐C_3_N_4_‐OH based membranes and corresponding cross‐sectional SEM images. b) Permeance and c) separation factor of the mixed acetonitrile/toluene solution for g‐C_3_N_4_‐OH membranes prepared with varied thickness and lateral size of nanosheets, respectively. Reproduced with permission.^[^ ^115^ ^]^ Copyright 2021, Royal Society of Chemistry.

More recently, Jiang et al. prepared a novel laminated membrane with controllable interlayer pathways via employing g‐C_3_N_4_ nanosheets as the building blocks.^[^ ^115^ ^]^ The g‐C_3_N_4_ nanosheets with various lateral sizes and thicknesses were edge‐functionalized by ‐OH groups and then assembled into lamellar membranes through vacuum‐assisted filtration process (**Figure** **16**a). It was believed that the cross‐layer channels could show a “gate effect” to control the molecular transport rate depending on their shapes and sizes. The results showed the optimized membrane exhibited an unprecedented acetonitrile permeance of 2128 L m^−2^h^−1^bar^−1^, along with the acetonitrile/toluene selectivity as high as 12.7 (Figure 16b,c). These expectations were hard to realize for the traditional polymer membranes.

### 4.4 Ionic Exchange/Transport

#### 4.4.1 Fuel Cells

With more attention being paid to fossil fuel depletion and environmental deterioration, fuel cells are becoming a promising option in terms of the utilization of renewable and clean energy. Ionic exchange/transport membranes are an important part acting as an electronic insulator and insulate oxygen and hydrogen. Because of the stable and organized framework structures, large surface areas, and designable pore sizes, 2D polymer nanosheets‐based membranes have enough space to accommodate proton/anion carriers, endowing them with tunable proton/anion conductivity, and thus holding great promise as the candidates of ionic exchange/transport membranes.

**Figure 17:**
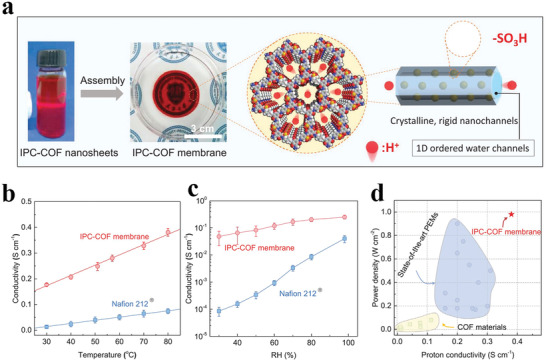
COF nanosheets used for proton exchange membranes. a) Scheme of the IPC‐COF membrane assembly process and the model of the pore structures. b) Proton conductivity of IPC‐COF membrane and Nafion 212 at different temperatures. c) RH‐dependent proton conductivity of IPC‐COF membrane and Nafion 212. d) Comparison of proton conductivity for IPC‐COF membrane, COF materials, and state‐of‐the‐art PEMs. Reproduced with permission.^[^ ^61^ ^]^ Copyright 2020, Wiley‐VCH.

For example, Jiang et al. prepared intrinsically proton‐conducting COF (IPC‐COF) nanosheets and then fabricated the IPC‐COF membranes by vacuum‐assisted filtration (**Figure** **17**a).^[^ ^61^ ^]^ Benefiting from the well‐ordered, rigid proton transport nanochannels and abundant proton carriers(‐SO_3_H), the obtained IPC‐COF membranes showed an unprecedentedly high proton conductivity, with a maximum value of 0.38 S cm^−1^ at 80 °C, which was almost 4 times higher than that of Nafion 212 (Figure 17b). Meanwhile, the relative humidity had little effect on the proton conductivity (Figure 17c), which was believed to be very suitable for proton exchange membrane‐based fuel cells in practical applications. The testing results showed that the obtained IPC‐COF membrane achieved the highest power density among the current COF materials and even superior to those of state‐of‐the‐art PEMs reported in the literature (Figure 17d).

**Figure 18:**
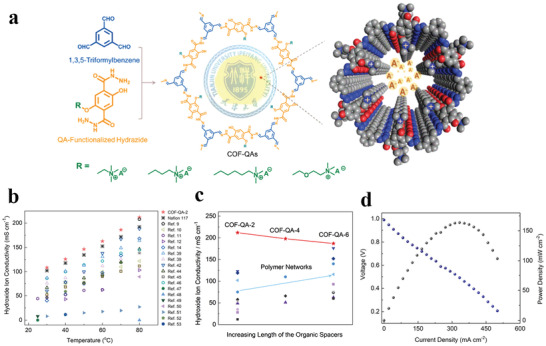
COF nanosheets used for anion exchange membranes. a) Schematic diagram for synthesizing COF‐QAs. b) Comparison in hydroxide ion conductivity of the COF‐QA‐2 membrane with other anion‐conducting materials. c) Relationship between hydroxide ion conductivity and length of the spacers from the COF‐QAs membranes and conventional polymer networks. d) Polarization and power density curves of the COF‐QA‐2 membrane in H_2_/O_2_ single cell tested at 60 °C, 100% RH. Reproduced with permission.^[^ ^116^ ^]^ Copyright 2020, Wiley‐VCH.

In addition to proton exchange membrane, 2D polymer nanosheets could also be utilized to assemble into anion exchange membrane. For example, in 2020, Jiang's group utilized organic‐aqueous interface reaction system to synthesize COF nanosheets and then assembled COF nanosheets into composite membranes for fuel cells applications (**Figure** **18**a).^[^ ^116^ ^]^ In order to construct anion transport pathways, hydrazide building units bearing quaternary ammonium (QA) groups with different side chains were designed and synthesized, which were then reacted with the aldehyde monomer to produce a series of side‐chain quaternized COFs (COF‐QAs). The as‐prepared membranes exhibited a high hydroxide conductivity (212 mS cm^−1^ at 80 °C), which was superior to those of the reported anion exchange membranes (Figure 18b). Meanwhile, it was found that the shorter, more hydrophilic side chains were more beneficial for anion transport (Figure 18c). In order to investigate the application potential of COF‐QA membranes in fuel cells, H_2_/O_2_ single fuel cell test was carried out at 60 °C. As shown in Figure 18d, the COF‐QA‐2 membrane obtained an open circuit voltage of 0.989 V together with a high‐power density of 163.7 mW cm^−2^. Based on the findings, the same group investigated the effect of size and hydrophilicity of aldehyde units on the formation of COF nanosheets, and the structures as well as the anion conductivity of the assembled membrane. It was found that the smaller and more hydrophilic aldehyde monomers helped to promote the interfacial reaction‐diffusion process, allowing for the controlled assembly of COF‐QA membranes with a tighter structure, which would result in a fast anion diffusion rate.^[^ ^117^ ^]^

#### 4.4.2 Lithium‐Sulfur Batteries

In addition to fuel cells, lithium‐sulfur batteries are also an important energy storage device. For high‐performance lithium‐sulfur batteries, ion selective membrane separators with rapid lithium‐ion transport and good polysulfides‐blocking capability remain important and highly sought. In lithium‐sulfur batteries, the successful technique of depositing a functionalized layer over the separator is commonly used to inhibit the diffusion of polysulfides. Given the benefits of large surface areas, uniform pore distribution, light weight, and durable durability, 2D polymer nanosheets may be utilized to construct a modified layer for blocking polysulfides.^[^ ^118^, ^119^ ^]^ For example, Sun's group synthesized 2D polymer nanosheets and subsequently deposited modified layers on separator to form composite membranes, which contained abundant phenolic and triazole units as well as lithiated‐sites in the ordered channels of polymer nanosheets.^[^ ^120^ ^]^ Thanks to the unique structure, the Li‐COF‐based membrane separator can considerably facilitate lithium‐ion transport while limiting polysulfide diffusion. As a result, the obtained cell exhibited higher specific capacities at various rates. Moreover, the cell exhibited good long cycling performance, high discharge capacity, and relatively little capacity decay.

**Figure 19:**
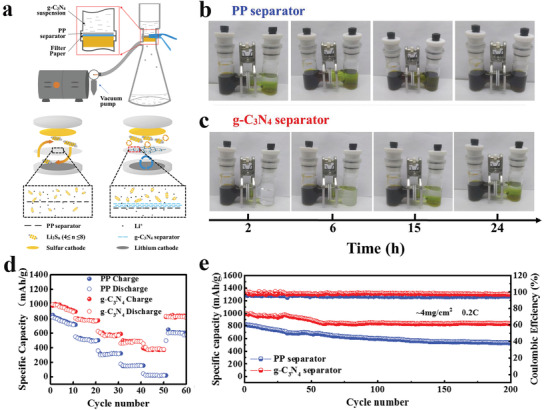
g‐C_3_N_4_ nanosheets used for the membrane separator in Li‐S batteries. a) The scheme of the systematic Li‐S batteries with g‐C_3_N_4_ separator. Visualized H‐type battery with b) PP separator and c) g‐C_3_N_4_ separator, respectively. d) Rate capabilities and e) cycle performance of the Li‐S batteries with g‐C_3_N_4_ separator. Reproduced with permission.^[^ ^121^ ^]^ Copyright 2018, Elsevier.

**Figure 20:**
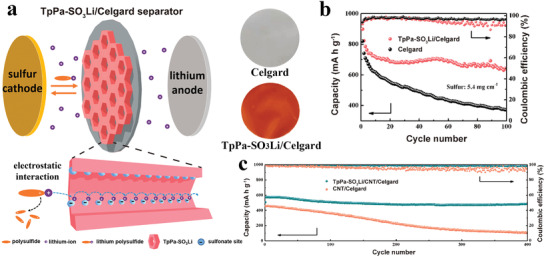
COF nanosheets used for the membrane separator in Li‐S batteries. a) Schematic of the Li‐S cell with the TpPa‐SO_3_Li/Celgard separator and optical photograph of Celgard and TpPa‐SO_3_Li/Celgard. b) Cycling performance at 0.2 C of the battery with TpPa‐SO_3_Li/Celgard or Celgard as the separator. c) Long‐term cycling performance of the battery. Reproduced with permission.^[^ ^122^ ^]^ Copyright 2021, American Chemical Society.

In another study, Xie et al.^[^ ^121^ ^]^ developed g‐C_3_N_4_ nanosheets‐based separator through a vacuum‐filtration method onto the commercial polypropylene (**Figure** **19**a). The dense structure of assembled g‐C_3_N_4_ film and enriched polysulfides adsorption sites of pyridinic‐N within C_3_N_4_ structure were thought to be useful to restrict the migration of polysulfides (Figure 19b,c). As a result, the battery with g‐C_3_N_4_ separator showed an initial specific capacity of 990 mA h g^−1^ and an initial Columbic efficiency of 99.8% at the current rate of 0.2 C. Moreover, the g‐C_3_N_4_ separator‐based battery exhibited a higher reversible capacity of 829 mA h g^−1^ after 200 cycles corresponding to capacity retention of 83.7%, which was much better than that of the traditional PP separator based battery (Figure 19d,e). This result demonstrated a great potential of g‐C_3_N_4_ separator for Li‐S batteries. More recently, Cao et al. fabricated an ion selective 2D polymer nanosheets‐based film (termed as TpPa‐SO_3_Li) on the commercial Celgard separator.^[^ ^122^ ^]^ Through electrostatic interaction, the oriented nanochannels and continuous negatively charged sites may effectively boost lithium‐ion conduction while minimizing polysulfide diffusion (**Figure** **20**a).^[^ ^123^ ^]^ Consequently, with the aid of TpPa‐SO_3_Li layer, the Li‐S battery achieved a high initial capacity of 822.9 mA h^−1^ g^−1^ and a good cycling stability (Figure 20b,c)

## 5 Conclusions and Outlooks

2D polymer nanosheets are ideal candidates for the design of high‐performance membranes for gas separation, water purification, organic solvent separation, and ionic exchange/transport owing to their high porosity, large surface areas, orderly pore structures, adjustable apertures, and facile functionalization. Although tremendous progress has been made in 2D polymer nanosheets‐based membrane separation technology, many challenges still remain and need serious consideration.

To date, most nanosheets preparation technologies were limited in the laboratory, usually suffering from the drawbacks of high‐cost, time‐consuming, and complicated synthetic steps. In order to achieve potential applications of 2D polymer nanosheets, it is highly desired to develop high‐efficiency and low‐cost strategies to produce high‐quality and large‐scale 2D polymer nanosheets. In the construction of composite membranes from ultrathin nanosheets, one of the major challenges is that membranes with sub‐nanometer pore sizes are difficult to achieve with precise ion/molecule sieving. Although the length of building blocks may be changed to regulate the form and size of pores ranging from nanopores to sub‐nanopores, pure 2D polymer nanosheets‐based membranes with effective pore diameters smaller than 1 nm are still uncommon owing to the building unit size constraint and the presence of nonselective defects. In this regard, pre/post‐treatment could be an effective strategy. Modified molecules could be anchored with the functional groups within the pore walls through covalent or metal‐coordination reaction so as to effectively reduce the membrane pore size down to sub‐nanometer scale. Besides, the mixed assembly of 2D polymer nanosheets with other polymeric materials could be utilized as an effective strategy to avoid the production of nonselective defects within nanosheets‐assembled membranes.

From the view point of the practical applications, in addition to perm‐selectivity, the mechanical strength and structural stability of 2D polymer nanosheets‐based membranes are also two key parameters needed to be carefully considered because the practical cross‐flow operation and various acid or alkaline environment could cause the loss or even decomposition of polymer sheets, which will result in the sharp decline of membrane separation performance. Therefore, it is very necessary to construct pH‐resistant 2D polymer nanosheets via the selection of suitable organic linkers with strong covalent bonds in terms of the molecular design. On the other hand, more attention should be paid on improving the interaction among polymer nanosheets and the adhesive strengths between nanosheets‐based layer and membrane substrate, so as to enhance the membrane structural stability. This could be accomplished by two kinds of strategies. The first one is to achieve covalent cross‐linking by using suitable chemical cross‐linkers that could react with the functional groups within the 2D polymer nanosheets. The more attractive strategy is to directly construct 2D polymer nanofilm on the supporting membrane through in situ interfacial polymerization, similar to the production approach of traditional polyamide‐based NF or RO membranes. Such strategy is facile to achieve large‐scale production avoiding the complex and time‐consuming assembly process of polymer nanosheets‐based membrane. Besides, it is worth noting that current research in this area is mainly focused on gas separation and mild liquid separation, including dye removal, water desalination, and so on. In harsh environments or sophisticated systems, the long‐term durability of 2D polymer nanosheets‐based membranes needs to be further explored.

In the end, although many challenges remain to be overcome, we hope to provide a comprehensive review of the research progress in the field of membrane separation based on 2D polymer nanosheets materials. Meanwhile, with fast developments in synthetic chemistry, material chemistry, and chemical engineering, membrane design concepts and separation applications will soon evolve into real world. Finally, we believe that this review would assist membrane researchers in better using the benefits of 2D polymer nanosheets.

## Conflict of Interest

The authors declare no conflict of interest.
